# Clustering the lexicon in the brain: a meta-analysis of the neurofunctional evidence on noun and verb processing

**DOI:** 10.3389/fnhum.2013.00303

**Published:** 2013-06-27

**Authors:** Davide Crepaldi, Manuela Berlingeri, Isabella Cattinelli, Nunzio A. Borghese, Claudio Luzzatti, Eraldo Paulesu

**Affiliations:** ^1^MoMo Lab, Department of Psychology, University of Milano-BicoccaMilan, Italy; ^2^Department of Psychology, University of Milano-BicoccaMilan, Italy; ^3^AIS Lab, Department of Computer Science, University of MilanoMilan, Italy

**Keywords:** neuroimaging, noun-verb dissociation, meta-analysis, clustering algorithm, task demand, left inferior frontal gyrus

## Abstract

Although it is widely accepted that nouns and verbs are functionally independent linguistic entities, it is less clear whether their processing recruits different brain areas. This issue is particularly relevant for those theories of lexical semantics (and, more in general, of cognition) that suggest the embodiment of abstract concepts, i.e., based strongly on perceptual and motoric representations. This paper presents a formal meta-analysis of the neuroimaging evidence on noun and verb processing in order to address this dichotomy more effectively at the anatomical level. We used a hierarchical clustering algorithm that grouped fMRI/PET activation peaks solely on the basis of spatial proximity. Cluster specificity for grammatical class was then tested on the basis of the noun-verb distribution of the activation peaks included in each cluster. Thirty-two clusters were identified: three were associated with nouns across different tasks (in the right inferior temporal gyrus, the left angular gyrus, and the left inferior parietal gyrus); one with verbs across different tasks (in the posterior part of the right middle temporal gyrus); and three showed verb specificity in some tasks and noun specificity in others (in the left and right inferior frontal gyrus and the left insula). These results do not support the popular tenets that verb processing is predominantly based in the left frontal cortex and noun processing relies specifically on temporal regions; nor do they support the idea that verb lexical-semantic representations are heavily based on embodied motoric information. Our findings suggest instead that the cerebral circuits deputed to noun and verb processing lie in close spatial proximity in a wide network including frontal, parietal, and temporal regions. The data also indicate a predominant—but not exclusive—left lateralization of the network.

## Introduction

Following the seminal report of a dyslexic patient who was predominantly impaired in reading verbs compared to nouns (Holmes et al., 1971), substantial evidence has been accumulated which supports the hypothesis that noun and verb representations are functionally independent in the human cognitive system. This evidence sources primarily from neuropsychological studies describing various patients whose behavior collectively supports the case for double noun-verb dissociation (Miceli et al., 1984; Hillis and Caramazza, 1995; Berndt et al., 1997; Luzzatti et al., 2002; Crepaldi et al., 2006), but is also confirmed by several psycholinguistic studies in which nouns and verbs give rise to different pattern of priming effects (Sereno, 1999; Mahon et al., 2007; Crepaldi, 2008).

The functional dissociation between nouns and verbs raised the question as to whether the neural underpinnings of these grammatical classes are anatomically segregated in separate brain regions. This issue was initially investigated in anatomo-correlational studies, which altogether revealed a somewhat controversial picture. Damasio and Tranel (1993), for example, reported the case of two patients who had suffered from temporal damage and whose ability to retrieve nouns was specifically impaired, and of one patient who had suffered a damage to the posterior segment of the inferior frontal gyrus and whose ability to retrieve verbs was impaired. In spite of some replication of this fronto-temporal pattern (Daniele et al., 1994), these results do not fit easily with what has been reported in several other anatomo-clinical studies. For example, Aggujaro et al. (2006) studied lesion localization in a sample of 20 aphasic patients suffering from disproportionate impairment of either nouns or verbs: they found no verb-impaired patient with a pure frontal damage, and several cases with isolated left posterior-temporal and inferior-parietal brain damage. Converging data arise from a study by De Renzi and Di Pellegrino (1995), who described an aphasic patient with vast frontal brain damage, but no specific problems in retrieving verbs.

Data from functional neuroimaging studies are also rather unclear as to whether the neural structures responsible for noun and verb processing are anatomically segregated in the brain. In one of the first neuroimaging investigations about this issue, Warburton et al. (1996) compared the cerebral activation related to nouns and verbs in a verbal fluency task: they concluded that the two grammatical classes recruit the same neural circuits, but verbs elicit stronger activations in these areas than nouns. In spite of early replications of these findings (Perani et al., 1999), several other studies have found that nouns and verbs do recruit spatially segregated brain regions or, conversely that the two grammatical classes elicit similarly strong activations in the same areas. Saccuman et al. (2006) for example, working with an fMRI picture naming study, found verb-specific activation in the left intra-parietal sulcus, in the right fusiform gyrus, and in the left cerebellum, while nouns determined an increased BOLD signal in the right cuneus and the right posterior cingulate cortex. However, Tyler et al. (2001) reported diametrically opposing results in a lexical decision and a semantic categorization task; in their study none of the cortical areas (with the sole exception of the left BA 20/37) was activated in direct verbs-minus-nouns or nouns-minus-verbs comparisons.

Results continue to be somewhat inconsistent if one considers the locations of verb- and noun-specific areas in those studies where grammatical class effects were actually found. For example, Shapiro et al. (2005) used a word/pseudo-word inflection task and found that verbs provoked greater activation than nouns in the anterior portion of the left superior frontal gyrus, in the LIFG including Broca's area, and in the right cerebellum, while nouns elicited stronger activation than verbs in the middle part of the superior temporal gyrus, the middle portions of the left fusiform gyrus, and in the right insula and cerebellum. These results are in line with the fronto-temporal dichotomy originally described by Damasio and Tranel (1993), and were further confirmed in other neuroimaging studies (Chao and Martin, 2000; Tranel et al., 2005a). However, no verb-specific frontal activation was found in other experiments. Damasio et al. (2001) for example, observed verb-specific activation in the middle left temporal gyrus in an experiment where picture naming was compared to a non-linguistic baseline (i.e., orientation judgment on unfamiliar faces). Berlingeri et al. (2008) conducted a factorial study with two experimental tasks (picture naming of nouns and verbs, and a verb-from-noun and noun-from-verb derivation task), and found reliable across-task verb-specific activation bilaterally in the precentral and postcentral gyri, in the right SMA, and again bilaterally in the paracentral lobule, the superior parietal lobule, the inferior parietal lobule, and the precuneus: none of the left dorsolateral prefrontal areas was activated to a greater extent by verbs than by nouns. Similar considerations can be made when we turn our attention to the brain areas that were shown to be associated to noun processing. Bedny and Thompson-Schill (2006) for example, found that the LIFG and the left inferior temporal gyrus were more strongly activated by nouns than by verbs in a semantic matching task. However, in a word inflection experiment Shapiro et al. (2006) found that the only area emerging from a direct nouns-minus-verbs comparison was the left fusiform gyrus.

These apparently inconsistent data are quite relevant for the hotly debated topic of sensorimotor contribution to abstract concept representation (e.g., Gallese and Lakoff, 2005) and, more in general, for that of embodied theories of cognition (e.g., Rizzolatti and Sinigaglia, 2010). In fact verbs typically denote actions, and frequently refer to human movements that clearly have motoric counterparts in the cognitive system (e.g., to walk, to pick, to throw, to talk); if indeed abstract concepts were truly based on sensorimotor knowledge, verb lexical-semantic representation would substantially call upon proprioceptive, tactile, and motoric information (e.g., Shebani and Pulvermüller, 2013). Several theories have been proposed based on this core idea. They range from a “soft” position whereby verb meaning relies on abstract representations that interact dynamically with our sensory and motor systems (Bedny and Caramazza, 2011), to a stronger position whereby the verb meaning itself is the sensory-motor experience that occurs every time a specific action is either made or observed (e.g., Pulvermüller, 1999; Gallese and Lakoff, 2005). Theories at the softer end of this continuum suggest that action verb processing relies on a wide network of amodal brain regions including left frontal, temporal, and parietal cortices; on the other hand, strong embodied views of cognition suggest that action verb processing is primarily based on the activity of the primary motor cortex (Hauk et al., 2004). Other scholars, working along similar lines, have reported data suggesting that verb processing relies on a network of action-related brain areas outside the motor strip (right SMA, right and left paracentral lobules, right and left superior and inferior parietal lobules, and right and left precuneus; Berlingeri et al., 2008), thus proposing that verb lexical processing activates action-oriented, visuo-spatial, rather than low-level motoric information. It is interesting to note that a clear divide between action verbs and non-action verbs does not emerge from these data (Aggujaro et al., 2006; Berlingeri et al., 2008) which would seem to imply that the parietal regions, which are the primary basis for the planning of object-related actions (Grefkes and Fink, 2005), are also involved in the lexical processing of non-action verbs.

Several other theories have been proposed to account for neurofunctional data on verb and noun processing. Originally, mostly on the basis of the influential paper by Damasio and Tranel (1993), verbs and nouns were held to have distinct and anatomically separate neural underpinnings, with verbs being mainly processed in the left frontal regions and nouns in the left temporal lobe. This position continued to be held for quite some time (Cappa et al., 2002; Cappa and Perani, 2003; Shapiro et al., 2006), but seems to be hardly tenable: as noted in a recent review by Crepaldi et al. (2011), of 15 neuroimaging studies that reported verb-noun direct contrasts, only five showed verb-specific activation in left frontal areas, and only two showed noun-specific activation in a left temporal region. Of course, caution should be taken when interpreting these figures, as the use of different technical and experimental details could determine changes in the results of fMRI studies (e.g., block vs. event-related design, statistical thresholds, sample size); but there still seems to be little justification for suggesting a specific role in verb processing for frontal areas. This consideration also casts doubts on a more recent proposal which suggests that verb-specific processing does not rely exclusively on frontal areas, but on a more complex circuit that includes the left middle frontal gyrus (Willms et al., 2011), or the temporo-parietal junction (Aggujaro et al., 2006; Tranel et al., 2008). Basically, any theory that attributes a substantial role to frontal areas in verb processing seems to be unsupported overall by fMRI/PET evidence (unless they can explain why these cerebral regions do not emerge as verb-specific in such a large proportion of the neuroimaging studies focusing on this issue).

Another popular position is that grammatical class *per se* is not an organizing principle in the neural organization of the language areas; rather, the main divide would be semantic, and would follow the object vs. action dichotomy (Bird et al., 2000, 2003; Vigliocco et al., 2011). From a behavioral point of view, children would start by learning lexical labels for prototypical objects and prototypical actions, and subsequently would discover that their distribution in sentences varies and that they sub-serve different communicational roles (object words denote, action words predicate). The grammatical classes of nouns and verbs would then be built on the basis of these cues, but the distinction between the two would remain strongly linked to their origins. This is why noun-verb neural effects emerge clearly only when prototypical nouns (i.e., object nouns) and prototypical verbs (i.e., action verbs) are investigated (Vigliocco et al., 2011). From an anatomic point of view, this theory is very similar to that outlined in the previous paragraph: action (verb) processing would rely more on a fronto-parietal network, whereas object (noun) processing would depend on inferotemporal structures. Although functionally speaking the theory is plausible and might be separated from its anatomical counterpart, much of the neuroimaging evidence provided so far does not support either a specific role for frontal areas in action word/verb processing or for temporal regions in object word/noun processing (Tyler et al., 2001; Tranel et al., 2005a; Liljeström et al., 2008; Crepaldi et al., 2011).

It should be apparent that the wealth of alternative accounts is at least partly motivated by the diversity of the experimental results reported so far. It is thus essential to try to distinguish unreliable observations from those with a solid experimental base, also taking into account the number of factors that may underlie inconsistent results across neuroimaging studies on nouns and verbs. These factors include, for example, the high heterogeneity of the experimental and baseline tasks used in the various studies. In fact, different tasks involve different cognitive processes, with two important consequences: first, as it is plausible that different cognitive processes are carried out in different parts of the cortex, it is unlikely that, for example, the semantic processing of verbs will recruit the same areas as the phonological processing of verbs. Moreover, nouns and verbs might be anatomically segregated at some cognitive stage (e.g., morphological analysis), but not at others (e.g., phonological encoding); since different tasks tap into different cognitive stages, it is not surprising that anatomical separation might emerge in, e.g., picture naming, but not in, e.g., lexical decision. Even when only focusing on neuroimaging experiments, evidence has emerged from tasks such as picture naming and syntactic judgment, lexical decision and generation of derived forms (e.g., “dealer” from “deal”), forced-choice semantic association and verbal fluency. Orthographic processing, lexical identification, semantic processing, syntactic planning and analysis, lexical selection, and phonological encoding are all processing stages that have been addressed very differently in different studies, through the use of different experimental tasks. Task diversity is thus clearly a factor that has contributed variability to this literature (e.g., Berlingeri et al., 2008).

Another important factor is cognitive processing load: some recent studies have reported convincing evidence that brain activations change substantially according to whether a specific combination of task and stimulus imposes a high cognitive demand, or is instead very easy and fast to process (Thompson-Schill et al., 1997; Snyder et al., 2007; Berlingeri et al., 2008). Scholars have recently started to take these factors into account while evaluating whether the data currently available can be explained satisfactorily within a theoretical account. However, they have come to somewhat different conclusions. Vigliocco et al. (2011) suggest that, once cognitive demand is taken into consideration, neuroimaging data on nouns and verbs can indeed be interpreted in a theoretical framework that sits noun processing within the inferior temporal cortex and verb processing within a network involving frontal and parietal areas. On the contrary, Crepaldi et al. (2011) deny the possibility that neuroimaging data on nouns and verbs can be accounted for satisfactorily within any theoretical framework that assumes spatially segregated neural substrates for the two grammatical classes. They also suggest that this holds even after task-specific and cognitive demand effects were taken into account. The authors propose that nouns and verbs are processed in neural circuits that do not overlap completely (or otherwise neuropsychological dissociations would never be possible), but are *not* clearly spatially segregated, at least at the spatial resolution normally considered in neuroimaging studies. Noun and verb circuits would be strictly interleaved with each other and dispersed in a complex network spanning virtually all over the brain. Thus, the emergence of grammatical class specific regions in fMRI studies would be highly variable and very much dependent on fine details concerning the task used, the specific stimuli selected, the methods of analysis, etc. [for converging evidence in this direction, see Liljeström et al. (2009) and Sahin et al. (2009)].

To sum up, data on the neural basis of noun and verb processing seem to be highly inconsistent, to the point that no general theory proposed so far appears to be able to explain an acceptable proportion of them. Descriptive reviews of this literature have driven different authors to different conclusions (Crepaldi et al., 2011; Vigliocco et al., 2011), thus calling for a more formal assessment of this issue. In the present study fMRI data on nouns and verbs were thus submitted to a quantitative and theory-blind meta-analysis with the aim of addressing the following questions: (i) are the neural circuits responsible for noun and verb processing spatially segregated in the brain? (ii) If there are specific cerebral areas for nouns and verbs, where are they located? (iii) Which theory of the neural processing of nouns and verbs is best supported by this picture? As clearly highlighted above, while addressing these questions it is necessary to take into account which cognitive task generated brain activations. We thus adopted a methodological approach that allows not only to assess to what extent any brain region is committed to either nouns or verbs, but also whether grammatical-class specificity depends on the experimental task, or rather holds independently of this factor^1^.

There are several methods available for formal meta-analysis of neuroimaging data, among which the most popular is probably Activation Likelihood Estimation (ALE; Turkeltaub et al., 2002). The logic behind this approach is simple, and yet very powerful. A spatial probability distribution is modeled for each activation peak included in the dataset of interest. The voxel-by-voxel union of these distributions is used as an activation likelihood map, which is then tested for statistical significance against randomly generated sets of foci. ALE was proven to be a reliable way of blending evidence from multiple studies (e.g., Turkeltaub et al., 2012) and was applied successfully to fields as diverse as motor learning (Bernard and Seidler, 2013), autism (Dickstein et al., 2013), and numbers and mental calculation (Arsalidou and Taylor, 2011). However, it was not suited for our purposes. In particular, ALE is not able to deal with design with multiple independent variables, and here we want to consider the role of *both* grammatical class (X1) *and* task (X2). ALE strategy in these cases would be to consider separate sets of foci for each combination of grammatical class and task (nouns in picture naming, verbs in picture naming, nouns in lexical decision, and so on), and run one meta-analysis for each of these sets. This strategy would be problematic for two reasons. First, it would face a serious power issue: the overall dataset would be divided into several subsets, which would imply running meta-analyses on a low number of peaks. Second (and most important), such an analysis would tell us whether any given area is specific for any X1–X2 combination, but it would not show *in a statistically supported manner* whether any area is specific for, e.g., nouns in picture naming and verbs in lexical decision, or nouns in semantic tasks and verbs in syntactic tasks. In formal terms, it would not be possible to assess the interaction between grammatical class and task. Because there is solid evidence that this type of interactions do arise when assessing grammatical class specificity in different tasks (e.g., Palti et al., 2007; Berlingeri et al., 2008), this would have been a serious limitation of the ALE procedure.

We thus resorted to hierarchical clustering to carry out the meta-analysis (Jobard et al., 2003), using in particular the algorithm designed by Cattinelli et al. (2013a) and previously adopted by Cattinelli et al. (2013b). This algorithm permits the identification of clusters from a data set of noun-related or verb-related activation peaks on the basis of a pre-defined spatial resolution criterion. At this stage, the algorithm was completely blind as to which grammatical class or experimental task was associated with each single peak: it simply grouped peaks that were spatially close. After the clusters were identified, the distribution of noun- and verb-specific peaks in each cluster was statistically assessed in order to understand whether it was significantly different from chance. A similar analysis was carried out to capture grammatical-class specificities that were task-dependent (e.g., peaks that were associated with nouns in a given task, but with verbs in another task). The important point to make here is that the procedure was completely data-driven, and the spatial contiguity of the activation peaks was evaluated without any theoretical bias, a condition which is virtually impossible to reach in descriptive meta-analyses (e.g., Crepaldi et al., 2011; Vigliocco et al., 2011)—where some degree of subjective evaluation of data coherence is inevitable—or in original experimental studies where the experimental paradigm is generally constructed to assess some specific theoretical tenet.

## Methods

### Data collection and preparation

The present meta-analysis is based on 36 neuroimaging studies investigating the neural basis of noun and verb processing using either PET or fMRI on adult subjects, published on peer-reviewed journals from 1996 to March 2011. The studies were selected according to the following procedure. We first ran two queries through the PubMed database using the following search keys: “nouns AND verbs AND fMRI” and “noun AND verbs AND PET.” The search keys were sought in all entry fields. These queries generated 64 and 15 entries, respectively. Because we were also interested in papers that only included either nouns or verbs, we ran other four queries through the same database searching for “noun AND fMRI,” “nouns AND PET,” “verbs AND fMRI,” and “verbs AND PET.” After removing duplicates, we were left with 164 records, which were then screened to exclude those studies that clearly did not satisfy the inclusion criteria as revealed by the title, keywords, or abstract. For example, several studies did include nouns and/or verbs as stimuli, but focused on cognitive issues outside the interest of this meta-analysis (e.g., mental images, syntax); other studies presented nouns and verbs in a connected text, thus triggering semantic and syntactic processing that clearly hinders any lexical interpretation of the results; other studies did not make use of functional imaging techniques (i.e., were purely behavioral or neuropsychological studies), or investigated special populations, such as deaf people, children, elderly people, or patients with brain injuries or some form of degenerative disease. Fifty-six studies survived the screening and were thus assessed more thoroughly. Among these 56, 20 were excluded because they did not report any of the following: (i) a simple effect analysis of nouns vs. a non–noun baseline; (ii) a simple effect analysis of verbs vs. a non-verb baseline; (iii) a direct comparison analysis of verbs vs. nouns; (iv) a direct comparison analysis of nouns vs. verbs. Region-of-interest analyses were not considered.

The main characteristics of the 36 experiments included in this meta-analysis are reported in Table 1.

**Table 1:** **List of the papers included in the present metanalysis**.

**Authors**	**Year**	**Technique**	**Design**	**Sample size**	***p*-value**	**Experimental task**
Warburton et al., 1996	1996	PET	Block	9	0.005	Word fluency
Kiehl et al., 1999	1999	fMRI	Block	6	0.001	Visual lexical decision
Perani et al., 1999	1999	PET	Block	14	0.001	Visual lexical decision
Friederici et al., 2000	2000	fMRI	Event	14	0.001	Syntactic task
Damasio et al., 2001	2001	PET	Block	20	0.05	Picture naming
Tyler et al., 2001	2001	PET	Block	9	0.05 (FWE)	Visual lexical decision and Semantic task
Grossman et al., 2002	2002	fMRI	Block	16	0.005	Semantic task
Hugdahl et al., 2003	2003	fMRI	Block	13	0.001	Auditory lexical decision
Tyler et al., 2003	2003	fMRI	Event	12	0.001	Semantic task
Davis et al., 2004	2004	fMRI	Event	12	0.05 (FDR)	Semantic task
Hernandez et al., 2004	2004	fMRI	Block	9	0.001	Syntactic task
Li et al., 2004	2004	fMRI	Block	8	0.001	Visual lexical decision
Rowan et al., 2004	2004	fMRI	Event	10	0.05 (FWE)	Word fluency
Tyler et al., 2004	2004	fMRI	Event	12	0.001	Semantic task
Shapiro et al., 2005	2005	PET	Block	12	0.001	Inflection task
Tranel et al., 2005a	2005a	PET	Block	10	0.05	Picture naming
Tranel et al., 2005b	2005b	PET	Block	10	0.05 (FWE)	Picture naming
de Diego Balaguer et al., 2006	2006	fMRI	Event	12	0.001	Inflection task
Marangolo et al., 2006	2006	fMRI	Block	10	0.01	Derivational task
Saccuman et al., 2006	2006	fMRI	Event	13	0.05 (FDR)	Picture naming
Shapiro et al., 2006	2006	fMRI	Event	10	0.005	Inflection task
Yokoyama et al., 2006	2006	fMRI	Block	28	0.05 (FDR)	Visual lexical decision
Grossman et al., 2007	2007	fMRI	Event	25	0.05 (FWE)	Semantic task
Longe et al., 2007	2007	fMRI	Event	12	0.001	Semantic task
Thompson et al., 2007	2007	fMRI	Event	17	0.05 (FDR)	Visual lexical decision
Berlingeri et al., 2008	2008	fMRI	Block	12	0.001	Picture naming and derivational task
Heim et al., 2008	2008	fMRI	Block	28	0.05 (FWE)	Word fluency
Liljeström et al., 2008	2008	fMRI	Block	15	0.001	Picture naming
Siri et al., 2008	2008	fMRI	Mini-block	12	0.05 (FDR)	Picture naming
Tyler et al., 2008	2008	fMRI	Event	15	0.001	Semantic task
Crescentini et al., 2010	2010	fMRI	Block	14	0.05 (FWE)	Derivational task
Finocchiaro et al., 2010	2010	fMRI	Event	16	0.001	Inflection task
Khader et al., 2010	2010	fMRI	Event	17	0.05 (Bonferroni)	Word fluency
Thompson et al., 2010	2010	fMRI	Event	17	0.05 (FDR)	Auditory lexical decision
van Dam et al., 2010	2010	fMRI	Event	16	0.005	Semantic task
Rodríguez-Ferreiro et al., 2011	2011	fMRI	Block	14	0.001	Semantic task

FWE, Family Wise Error correction for multiple comparisons; FDR, False Discovery Rate correction for multiple comparisons.

We considered peaks emerging from simple effects of nouns vs. baseline and verbs vs. baseline, and peaks corresponding to direct comparisons of verbs-minus-nouns and nouns-minus-verbs; activation coordinates that emerged in conjunction analyses or main effects (e.g., the main effect of task irrespective to grammatical class) and those reflecting more selective processes (e.g., pure morphological processes, i.e., inflection of regular verbs vs. inflection of irregular verbs) were excluded.

After applying the above criteria the final working dataset was composed of 946 stereotaxic activation loci, 454 associated with nouns and 492 associated with verbs. Activation peaks were also classified according to the experimental task in which they were generated. We considered as separate categories in this variable: (i) lexical decision; (ii) semantic tasks (including semantic categorization tasks, forced-choice semantic association tasks, pleasantness judgment tasks, and synonym monitoring tasks); (iii) picture naming; (iv) generation tasks (including classical fluency tasks and cued single-item generation); (v) derivational tasks; (vi) inflectional tasks, including morphological judgment and phrase completion, when this required the subjects to generate the correctly inflected form; and (vii) syntactic judgment tasks. We did not separate tasks on the basis of whether they required covert vs. overt responses; however, in the majority of the experiments considered in this work participants were required to produce their responses covertly, so as to avoid movement-related artifacts in the imaging data.

The stereotaxic coordinates of earlier studies—in which activation peaks were reported in terms of the Talairach and Tournoux atlas (Talairach and Tournoux, 1988)—were transformed into the more recent MNI (Montreal Neurological Institute) stereotaxic space (Mazziotta et al., 1995); the transformation was done using a MATLAB script described at http://imaging.mrc-cbu.cam.ac.uk/imaging/MniTalairach.

### Clustering procedure

Functions available with MATLAB 7 (MathWorks corporation, 2004) were used to execute hierarchical clustering of activation peaks. The code is available from the third author on request.

**Figure 1:**
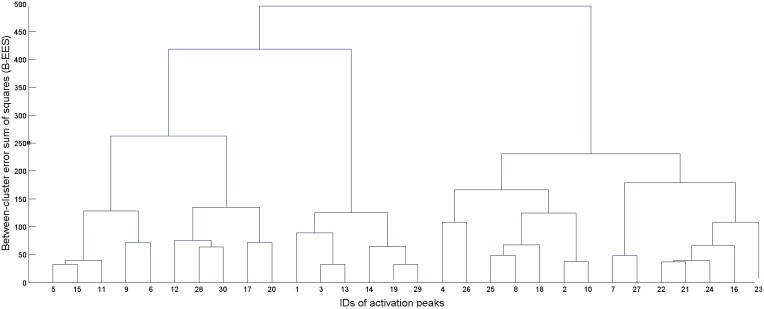
**Example of dendrogram (tree) resulting from the hierarchical clustering procedure**. The leaves at the bottom represent each individual activation coordinate. At each subsequent step, two clusters from the level immediately below are merged to form a new cluster. The number of clusters is thus decreased by one at each level, going from a total of *N* clusters at step 1 (where *N* is the number of input activation peaks) to one all-inclusive cluster at the last step.

First, the algorithm computed squared Euclidean distances between each pair of input data, and then merged, at each processing step, the two existing clusters with minimum dissimilarity. Dissimilarity was measured adopting Ward's (1963) criterion, which at each processing step selects the two clusters which, when merged, produce the minimum increase in the total intra-cluster variance. This procedure resulted in a tree (see Figure 1), whose leaves represent singletons (i.e., clusters formed of a single activation peak), and whose root represents one large cluster including all the 946 activation peaks input to the algorithm. Each level of the tree reports the clusters created by the algorithm at a specific processing step, as it progresses from individual activation peaks at the lowest level to the all-inclusive final cluster at the top of the tree. To determine the final set of clusters for further analyses (i.e., the level at which we “cut” the cluster tree), we averaged standard deviations in the *x, y*, and *z* directions over all clusters for each processing step. Starting from the leaves, we moved up the tree until the average standard deviation in each direction remained below 5 mm: this was done in order to obtain clusters whose dispersion around the center is compatible with a standard neuroimaging spatial resolution of approximately 10 mm.

Hierarchical clustering is sensitive to the order in which the individual peaks are processed, thus generating alternative clustering trees (Morgan and Ray, 1995). In order to tackle this problem and preserve the uniqueness of the clustering solution, a variant of the original algorithm was used which considers all different clustering solutions (given a specific spatial resolution) and attempts to identify the best one on the basis of their between-cluster error sum of squares (*B*-*EES*), defined as:

(1) $$B-EES=\sum_{k\;=\;1}^{C}n_{k}{\left(\mu_{k}-\mu_{X}\right)}^{2}$$

where *C* is the number of clusters in the considered solution, *n_k_* is the number of elements in the cluster *k*, μ_*k*_ is the mean of the cluster *k*, and μ_*X*_ is the mean of the entire dataset. Basically, *B – EES* quantifies the spatial separation between the clusters, and the best clustering solution is considered to be the one with maximal separation, i.e., maximal *B* – *EES*.

The mean coordinates of each cluster included in the final set were then passed as an input to a MATLAB script that was developed for the automatic anatomical labeling of the activation coordinates. This script queries the Automatic Anatomical Labeling (AAL) template available in the MRIcro visualization software (Rorden and Brett, 2000) to identify each individual cluster on the basis of its mean coordinates.

Hierarchical clustering identifies clusters of stereotaxic coordinates on the grounds that the resulting solution (the set of resulting clusters and the sets of coordinates that compose each cluster) has a minimized within-cluster and between-cluster variance. This procedure, as discussed in the Introduction, has the advantage of permitting a *post-hoc* assessment of the functional meaning of a given cluster on the basis of its data content. However, it does not quantify the significance of each individual cluster with reference to the probability of a spatially distributed statistical process. This aspect was investigated further by checking that our significant clusters would have also emerged with a different meta-analytical method, i.e., the Activation Likelihood Estimate as implemented in the GingerAle software^2^ (Eickhoff et al., 2009; Turkeltaub et al., 2012).

### Statistical analysis

**Figure 2:**
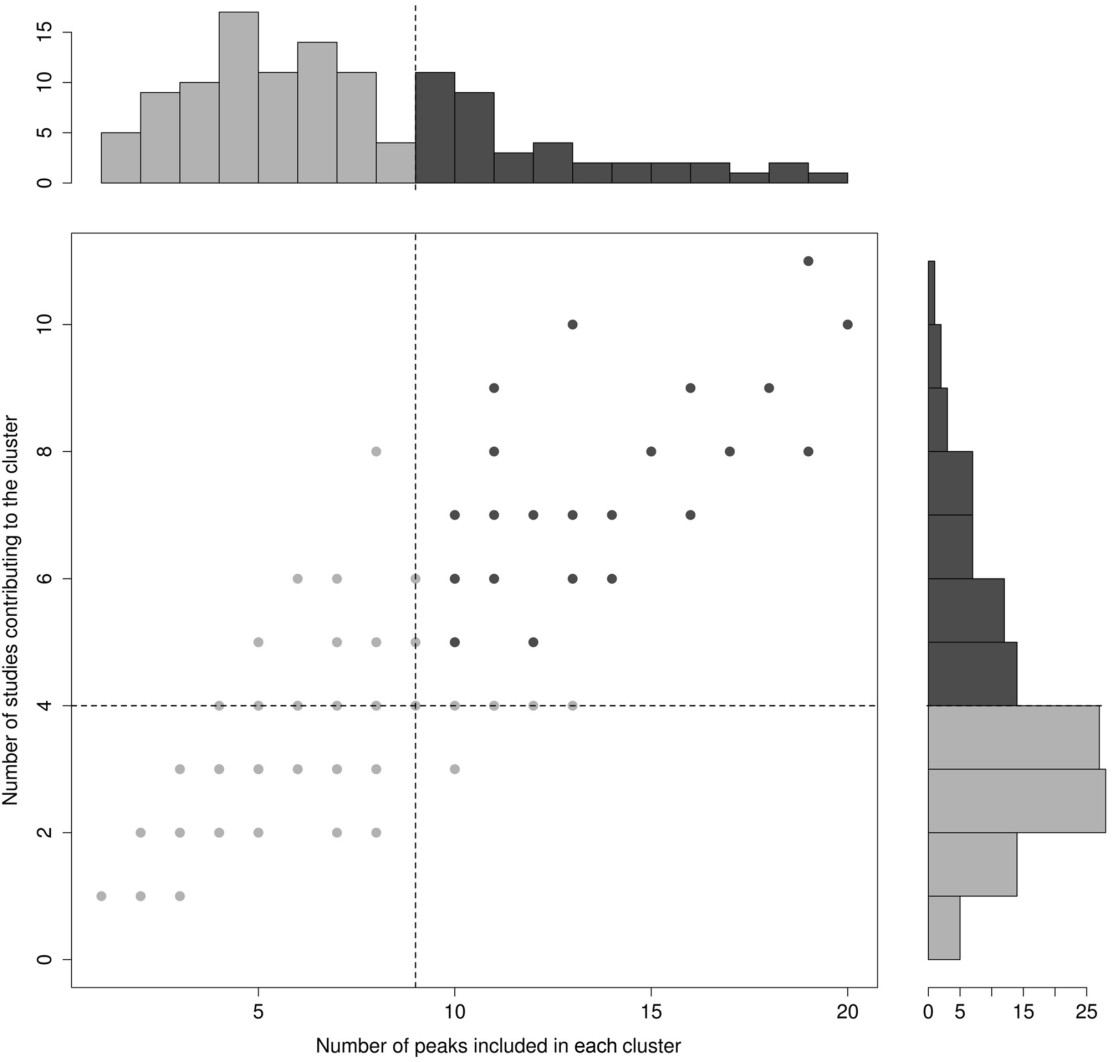
**Cluster distribution for the number of peaks included in each cluster (*X*-axis) and the number of studies contributing peaks to each cluster (*Y*-axis)**. The bimodal distribution of these variables is illustrated in the main panel, where each point represents a cluster (note that several points/clusters may overlap because of a same number of studies and peaks included). Unimodal distributions are represented through the histograms in the side panels. Dotted lines represent cut-off values.

In order to guarantee sufficient statistical power to the analyses and to exclude clusters that were not clear sign of converging evidence, only those clusters that contained 10 or more activation peaks, coming from at least five different studies were considered further. Because it was impossible to determine *a priori* the exact cluster size that granted the statistical analysis the desired reliability, the 10-peaks and 5-studies thresholds were set *a posteriori* on the basis of the actual distribution of the relevant variables in the final cluster set (see Figure 2).

The clusters that survived this selection were analysed in order to assess whether they were associated with (i) either grammatical class, or (ii) a specific task, or (iii) showed a more complex pattern reflecting a task-by-grammatical-class interaction. For each cluster, we created a contingency table reporting the number of activation peaks for each combination of grammatical class (verbs vs. nouns) and experimental task (lexical decision tasks vs. semantic tasks vs. picture naming vs. fluency tasks vs. inflectional tasks vs. derivational tasks vs. syntactic tasks). To assess specificity for grammatical class, we tested whether the distribution of noun- and verb-related peaks within each cluster was significantly different from the overall proportion of noun- and verb-related peaks included in the whole sample of coordinates (492/946 = 0.52 for verbs and 454/946 = 0.48 for nouns). To this end, we used the binomial distribution and computed the probability of observing a specific number of peaks associated with a given grammatical class as the number of successes in a series of independent randomly-distributed trials: when this probability was below 0.05, the cluster was considered to be associated with either noun or verb processing. The same logic was applied to investigate task specificity; an exact multinomial test was used to compare the peak distribution by task within each cluster with the overall distribution of the entire set of peaks included in this meta-analysis. Task-by-grammatical class interaction was tested with Fisher's exact test (Fisher, 1970); this estimates whether the distribution of one categorical variable (grammatical class, in our case) varies according to the levels of a second categorical variable (experimental task), thus revealing clusters that were associated with either grammatical class in one task (e.g., nouns in morphological tasks), but with the opposite grammatical class in another task (e.g., verbs in picture naming). All post-clustering statistical analyses were performed using the free statistical software R (version 2.10.1; R Development Core Team, 2005); the code is available from the first author on request.

## Results

The algorithm identified a total of 120 clusters scattered all over the brain, with 1 to 20 individual peaks each, from 1 to 11 different studies (see Figure 2), and had mean standard deviations along the three axes of 4.41 mm (*x*-axis), 4.76 mm (*y*-axis) and 4.89 mm (*z*-axis). Thirty-two of these clusters included 10 or more peaks from at least 5 different studies and were thus analysed for grammatical class and task specificity, and for task-by-grammatical class interaction. A complete list of these clusters is provided in Table 2.

**Table 2:** **List of the clusters that include at least 10 activation peaks from at least five different studies obtained through the application of the algorithm**.

**ID**	**Center**			**Standard deviation**			**No. of peaks**	**No. of studies**	**Brain area**
	***X***	***Y***	***Z***	***X***	***Y***	***Z***			
**FRONTAL AREAS**									
71	−47	3	40	4.27	3.62	5.59	17	8	Left precentral gyrus
68	52	12	31	5.07	7.38	6.04	11	8	Right precentral gyrus
69	−7	7	53	2.5	3.72	4.65	15	8	Left SMA
16	−5	16	45	3.9	6.15	3.55	14	6	Left SMA
18	−44	32	14	5.57	2.62	4.41	13	10	Left IFG, pars triangularis
46	−48	25	26	3.99	3.3	4.54	19	8	Left IFG, pars triangularis
56	−45	17	9	3.18	4.13	3.3	13	6	Left IFG, pars triangularis
118	38	28	26	3.04	6.85	6.13	12	7	Right IFG, pars triangularis
72	−32	27	−5	4.02	3.1	4.9	11	6	Left IFG, pars orbitalis
100	−47	26	6	2.85	4.73	5.27	14	7	Left IFG, pars orbitalis
45	−54	15	19	3.75	3.43	2.82	10	6	Left IFG, pars opercularis
111	−43	11	29	4.18	4.15	4.21	20	10	Left IFG, pars opercularis
44	47	17	5	5.24	4.24	5.04	11	7	Right IFG, pars opercularis
57	−44	5	9	6.01	4.7	3.52	11	6	Left insula
73	−34	19	5	3.88	3.53	4.38	19	11	Left insula
42	33	22	−2	3.68	4.98	5.08	15	8	Right insula
**TEMPORAL AREAS**									
101	−51	10	−5	4.57	3.43	5.01	17	8	Left Superior temporal pole
6	−54	−54	−1	4.52	3.64	5.31	11	9	Left middle temporal gyrus
20	−60	−34	3	3.07	4.86	2.72	10	7	Left middle temporal gyrus
82	53	−36	3	2.55	4.45	4.3	10	5	Right middle temporal gyrus
64	−41	−49	−25	4.68	4.5	3.77	18	9	Left fusiform gyrus
116	−31	−34	−22	5.96	4.61	5.4	16	9	Left fusiform gyrus
**PARIETAL AREAS**									
94	−52	−12	43	3.35	5.23	4.38	11	6	Left postcentral gyrus
51	−31	−51	51	4.62	5.09	3.87	10	6	Left inferior parietal lobule
85	−44	−48	50	3.8	5.19	7.34	10	7	Left inferior parietal lobule
86	−47	−33	45	3.66	5.1	5.28	13	7	Left inferior parietal lobule
33	−33	−61	42	3.47	4.52	3.67	10	6	Left angular gyrus
**OCCIPITAL AREAS**									
2	−50	−71	4	3.25	3.02	4.74	16	7	Left middle occipital gyrus
32	−23	−93	−2	3.68	3.9	6.97	11	6	Left inferior occipital gyrus
40	−43	−71	−12	5.95	5.66	2.67	10	5	Left inferior occipital gyrus
**OTHER AREAS**									
66	−13	−18	13	3.91	3.47	3.43	10	5	Left thalamus
10	42	−53	−26	4.43	4.5	4.94	12	5	Right cerebellum

From left to right: cluster ID; mean X, Y, and Z coordinates of the peaks included in each cluster, and their standard deviation along the three axes; number of peaks included in each cluster; number of studies from which these peaks come; and brain area in which the central coordinates of each cluster are included.

**Figure 3:**
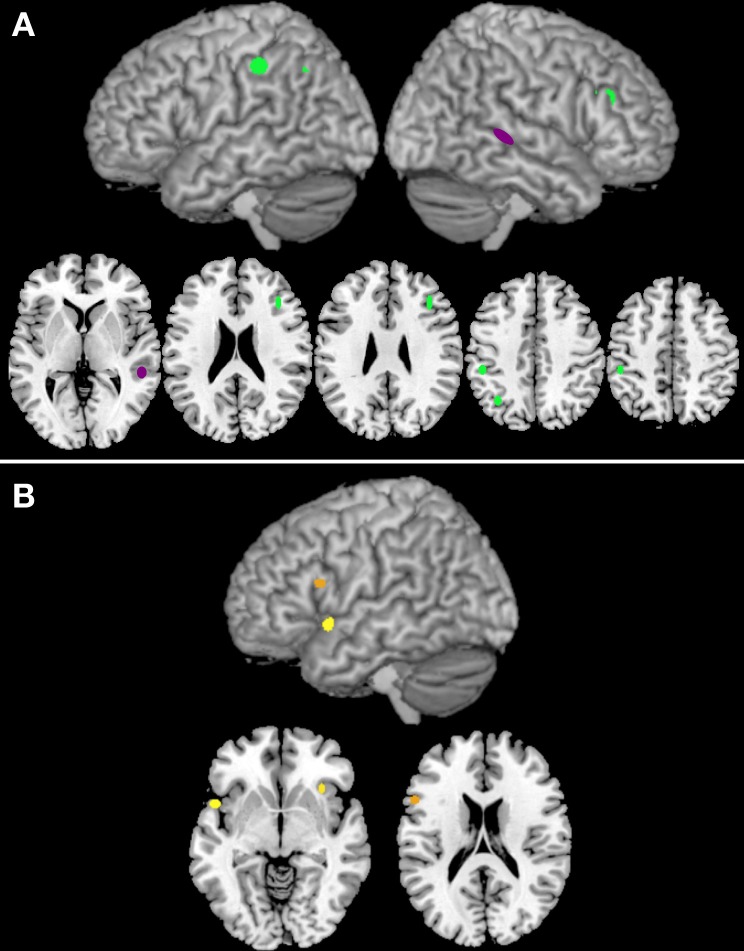
Panel **(A)** illustrates the clusters that are associated either with noun (green) or verb (purple) processing. Panel **(B)** reports the clusters that are associated with task-by-grammatical class interaction (the brighter the color, the higher the number of activation peaks included in the cluster).

Three clusters were associated with nouns (clusters 33, 86, and 118; see Table 3A), while only one was associated with verbs (cluster 82; Table 3B). The clusters associated with nouns were located in the left inferior parietal gyrus, the left angular gyrus, and the right inferior frontal gyrus, pars triangularis (see Figure 3A). The cluster associated with verbs was located in the posterior part of the right middle occipital gyrus (Figure 3A).

**Table 3:** **Noun- and verb-specific clusters as revealed by the meta-analytic procedure**.

**ID**	**Brain area**	**Peak distribution**		***p*-value**	
		**Nouns**	**Verbs**	**Nouns**	**Verbs**
**(A) NOUN-RELATED CLUSTERS**					
33	Left angular gyrus	8	2	0.04	
86	Left inferior parietal gyrus	12	1	0.001	
118	Right inferior frontal gyrus, pars triangularis	11	1	0.002	
**(B) VERB-RELATED CLUSTERS**					
82	Right middle temporal gyrus	1	9		0.05

From left to right: cluster ID; brain area in which the center of the cluster falls; noun- and verb-related peak distribution; probability associated with this distribution, calculated separately for noun and verb on the basis of a binomial distribution.

Three clusters (42, 45, and 101) showed task-by-grammatical class interaction as revealed by Fisher's exact test; these clusters were located in the right insula, in the left inferior frontal gyrus, pars opercularis, and in the left insula/left temporal pole (Figure 3B). The task-by-grammatical class distribution of the activation peaks included in each cluster is provided in Table 4.

**Table 4:** **Task-by-grammatical class distribution of the activation peaks included in each of the five clusters showing interaction between these two factors**.

	**Task**	**Nouns**	**Verbs**		***p*-value**
**ID = 42**	**Right insula**				
	LexDec	5	1		
	SemJdg	1	4		
	PicNam	0	1		
	Flu	0	0		
	Der	0	0		
	Infl	0	0		
	SyntJdg	3	0		
	Total	9	6		*p* = 0.048
**ID = 45**	**Left inferior frontal gyrus, pars opercularis**				
	LexDec	2	0		
	SemJdg	0	0		
	PicNam	0	0		
	Flu	0	4		
	Der	2	1		
	Infl	1	0		
	SyntJdg	0	0		
	Total	5	5		*p* = 0.048
**ID = 101**	**Left insula/Left temporal pole**				
	LexDec	1	1		
	SemJdg	0	2		
	PicNam	1	0		
	Flu	0	2		
	Der	1	0		
	Infl	0	1		
	SyntJdg	8	0		
	Total	11	6		*p* = 0.02

LexDec, lexical decision; SemJdg, semantic judgment; PicNam, picture naming; Flu, fluency tasks; Der, derivational tasks; Infl, inflectional tasks; SyntJdg, syntactic judgment.

Because task specificity was not the focus of this study and was only investigated as a co-varying variable for grammatical class (i.e., we were interested in the interaction between task and grammatical class, not in task effects *per se*), we do not report extensively on these results here, nor we will comment on them in the Discussion. However, we do note that the data on cluster task-specificity confirmed the reliability of our methodology, as they highlighted all the benchmark associations between tasks and brain areas, including fluency and the inferior frontal gyrus bilaterally (clusters 44 and 45); morphology and the left inferior frontal gyrus (clusters 100 and 111); word production in general (fluency + picture naming + derivational task) and the left inferior frontal gyrus (cluster 46); and picture naming and the middle occipital gyrus (cluster 2; see Table 5).

**Table 5:** **Task-specific clusters**.

		**Peak distribution**							
**ID**	**Brain area**	**LexDec**	**SemJdg**	**PicNam**	**Flu**	**Der**	**Infl**	**SynJdg**	***p*-value**
**OVERALL DISTRIBUTION IN THE WHOLE DATASET (*N* = 946)**									
		191 (20.2%)	142 (15%)	235 (24.8%)	139 (14.7%)	122 (12.9%)	68 (7.2%)	49 (5.2%)	
**(A) LEXICAL DECISION**									
46	Left inferior occipital gryus	*6 (60%)*	3 (30%)	0	0	1 (10%)	0	0	0.024
**(B) SEMANTIC JUDGMENT**									
16	L SMA	1 (7.1%)	*9 (64.3%)*	3 (21.4%)	0	0	0	1 (7.1%)	0.001
56	L IFG, pars triangularis	0	*7 (53.8%)*	3 (23.1%)	2 (15.4%)	0	0	1 (7.7%)	0.008
**(C) PICTURE NAMING**									
2	L middle occipital gyrus	0	1 (6.3%)	*14 (87.5%)*	1 (6.3%)	0	0	0	<0.001
116	L fusiform gyrus	1 (6.3%)	1 (6.3%)	*10 (62.5%)*	0	0	4 (25%)	0	0.001
**(D) FLUENCY**									
44	R inferior frontal gyrus	2 (18.1%)	3 (27.3%)	0	*6 (54.5%)*	0	0	0	0.008
45	L inferior frontal gyrus, pars opercularis	2 (20%)	0	0	*4 (40%)*	3 (30%)	1 (10%)	0	0.039
69	L SMA	2 (13.3%)	1 (6.7%)	1 (6.7%)	*6 (40%)*	3 (20%)	0	2 (13.3%)	0.037
**(E) DERIVATIONAL TASKS**									
100	L inferior frontal gyrus, pars orbitalis	0	2 (14.3%)	4 (28.5%)	1 (7.1%)	*6 (42.9%)*	0	1 (7.1%)	0.032
**(F) SYNTACTIC JUDGMENT**									
101	L superior temporal pole	2 (11.8%)	2 (11.8%)	1 (5.9%)	2 (11.8%)	1 (5.9%)	1 (5.9%)	*8 (47%)*	<0.001
**(G) CLUSTERS RELATED TO MORE THAN ONE TASK**									
42	R insula	*6 (40%)*	*5 (33.3%)*	0	1 (6.7%)	0	0	*3 (20%)*	0.001
46	L inferior frontal gyrus, pars triangularis	0	2 (10.5%)	*8 (42.1%)*	*5 (26.3%)*	*4 (21%)*	0	0	0.046
111	L inferior frontal gyrus, pars opercularis	0	3 (15%)	6 (30%)	3 (15%)	*4 (20%)*	0	*4 (20%)*	0.015

From left to right: cluster ID; brain area in which the center of the cluster falls; cluster distribution for task, in number of peaks and percentages; probability associated with this distribution, calculated on the basis of an exact multinomial test. Italic figures indicate the task where the majority of the peaks lie.

As a final assessment of our data, we evaluated its spatial significance with GingerAle, which showed that all seven clusters identified by hierarchical clustering and associated with either noun processing, verb processing or task-by-grammatical class interaction were also significant with this analysis (*p*_FDR_ < 0.05; Laird et al., 2005).

## Discussion

At a first glance, the results reported in the previous studies on the cerebral localization of noun and verb processing appear to be largely inconsistent (Perani et al., 1999; Tyler et al., 2001; Cappa et al., 2002; Saccuman et al., 2006; Siri et al., 2008; Crepaldi et al., 2011), which puts into question the generality of any theory proposed so far on the issue (Hauk et al., 2004; Berlingeri et al., 2008; Bedny and Caramazza, 2011). The present study aimed at providing a formal assessment of these apparently inconsistent data, so as to understand whether noun and verb processing recruits separate brain circuits, and, if so, where noun- and verb-specific areas are located in the human brain. We addressed these issues by feeding a clustering algorithm with all the activation peaks reported as grammatical-class specific in PET and fMRI studies on nouns and verbs; the clusters singled out by this process were then analysed to find out (i) whether they contained more noun- or verb-related peaks than might be expected on the basis of chance and (ii) whether the noun-verb distribution of the activation peaks varied across different experimental tasks. Three of the 32 reliable clusters generated by the algorithm were found to be associated with nouns; these clusters are located in the left inferior parietal gyrus, the left angular gyrus, and the right inferior frontal gyrus, pars triangularis. One cluster, located in the right middle occipital gyrus, included a higher-than-chance proportion of verb-related peaks. Finally, three clusters showed a task-by-grammatical class interaction; these were located in the right insula, in the left inferior frontal gyrus, pars opercularis, and in the left insula/left temporal pole.

A first, self-evident, observation is that the vast majority of the clusters identified by the algorithm (27 out of 32) is *not* specific for grammatical class. It is important to note that the peaks in these clusters have been included in this meta-analysis only because they were reported as either noun or verb specific *in the original studies*. In this perspective, the results suggest that brain areas may be specific for grammatical class in one particular study, but turn out to be not specific for grammatical class if all the data available is considered. The evidence just does not add up, confirming the results that emerged from a qualitative review (Crepaldi et al., 2011). Importantly, some of these clusters are located in areas that have been the focus of discussion in previous neuroimaging studies (e.g., the left inferior frontal gyrus, the left insula, the middle temporal gyrus) and that most scholars in the field consider as associated with either noun or verb processing as a matter of fact: this data utterly show that this is far from being clear.

This has important consequences for most of the theories on the neural underpinnings of noun and verb processing. Clearly, a position whereby frontal areas are predominantly involved in verb processing, whereas temporal regions are more active for noun processing (Damasio and Tranel, 1993; Cappa and Perani, 2003) is not tenable. No single verb-related cluster emerged in the frontal lobe, in spite of the fact that 17 clusters were identified in that brain region. Similarly, among the six clusters singled out by the algorithm in the left and right temporal lobes, none included significantly more noun peaks than verb peaks. Although radically different from the functional point of view, also the semantic theory of noun and verb representation put forward by Vigliocco et al. (2011) suggests that verb/action word processing should rely predominantly on frontal areas and noun/object word processing relies predominantly on temporal regions. This hypothesis is therefore not supported by the results of the present study.

Similar considerations can be made with respect to strong embodied views according to which lexical processing of movement verbs should elicit activation in the portion of the motor strip that represents the body part involved in the actual movement (e.g., the hand for “pick,” the tongue for “lick”; Hauk et al., 2004). As all types of verbs were considered in our meta-analysis, it was clearly unrealistic to expect that most verb clusters would be located in the primary motor area. However, six studies in our database employed motor verbs (Damasio et al., 2001; Grossman et al., 2002; Tyler et al., 2003; Saccuman et al., 2006; van Dam et al., 2010; Rodríguez-Ferreiro et al., 2011), which contributed 59 peaks to our set. Even considering that the motor verbs used in these studies involved different body parts (and would thus be expected to drive activation in different parts of the motor strip according to Hauk et al., 2004), it is surprising that none of these 59 peaks clustered into the primary motor area.

Bedny and Caramazza (2011) and Berlingeri et al. (2008) interpreted their results as indicating that verb processing is based on a fairly wide neural network, rather than on individual brain areas: the former authors suggested that this network is left lateralized and includes frontal, parietal, and temporal cortices, whereas the latter authors described data pointing to a more bilateral circuit, based particularly on posterior parietal areas. These reports do not seem to fit the data which emerged from this meta-analysis. None of the five parietal clusters identified by the algorithm were significantly associated with verbs (against Berlingeri et al., 2008). With regard to Bedny and Caramazza, (2011) proposal [but see also Hagoort (2005) and Mahon and Caramazza (2008)], the high rate of non-specific clusters is not compatible with a wide, verb-specific circuit that involves left frontal, temporal and parietal areas. Moreover, the location of those clusters that did show specificity for either grammatical class is also inconsistent with this view. It is possible, of course that a fronto-temporo-parietal network is indeed operating when decoding verbs (particularly action verbs), as suggested by Bedny and Caramazza (2011). However, this network is clearly also called upon by noun processing.

Altogether, the rigorous, theory-blind meta-analytic procedure used in this study confirms that the theories proposed so far are able to account for a limited portion of the available results. Moreover, they indicate that this does not depend on confounding variables. For example, several scholars have noted that the type of experimental task may affect which brain areas emerge as related to noun or verb processing (Palti et al., 2007); so, using different tasks in different studies may have hindered factual regularities in anatomo-functional correlations. However, we did take task into account in this study, and still the evidence remains weak for consistent associations between brain areas and grammatical classes (see also Crepaldi et al., 2011).

The vast predominance of unspecific clusters is more compatible with a framework in which a set of brain areas (including, but not limited to, the left inferior frontal gyrus, the left insula, and the middle temporal gyrus) is responsible for *both* noun *and* verb processing. The neural circuits related to these grammatical classes would be spatially segregated (otherwise neuropsychological dissociations and the grammatical-class specificity that emerged in certain imaging studies would never have been possible), but would also be located within the same brain areas, so as to become consistently separable only at a spatial resolution below those of fMRI and PET (Crepaldi et al., 2011).

Not only does this theoretical position reconcile the scattered neuroimaging evidence on noun and verb processing, but it is also strongly supported by three elements. The first emerged from our interaction analyses, which revealed that the left inferior frontal gyrus and the left insula, often assumed to be verb areas, are in fact associated with either noun or verb processing according to the specific task under investigation (see also Thompson-Schill et al., 1997; Berlingeri et al., 2008). Although this evidence should be treated with caution, given that some tasks were clearly under-represented in our set, it is in line with the hypothesis that these brain areas host both noun and verb related circuits, which are used in different ways by different test settings. The second and third elements in support of this “spatial-contiguity” hypothesis come from recent studies using imaging methods other than PET and fMRI: these studies demonstrated how noun- and verb-related cerebral activity are closely linked, both spatially and temporally. In an experiment comparing fMRI and MEG, Liljeström et al. (2009) failed to find any specific noun- or verb-related activation, with the only exception of a quasi-significant difference in the frontal region between 320 and 800 ms after stimulus presentation, i.e., well-below the temporal resolution allowed by fMRI. On the other hand, Sahin et al. (2009), using a methodology that combines a millisecond temporal resolution and a millimeter spatial resolution (Intra-Cranial Electrophysiology), showed that cortical signatures of lexical, syntactic and phonological processing for nouns and verbs are virtually identical, even in time windows that are well-below fMRI temporal precision (between 200 and 450 ms from stimulus presentation).

This general view of the neural underpinnings of noun and verb processing would also account for anatomo-correlative data. The neurophysiology of brain lesions clearly does not permit anatomo-clinical associations at a fine-grained spatial resolution: only sizeable lesions yield neuropsychologically relevant symptoms, so it is not possible to associate specific cognitive operations to particularly small brain regions. It follows that if noun and verb circuits are located close to each other in a specific brain area and can only be distinguished well below the spatial resolution allowed by anatomo-clinical correlation studies, it is not surprising that even similar brain lesions give rise to different behavioral patterns (e.g., a severe verb-specific impairment in one case—Damasio and Tranel, 1993; Tranel et al., 2008—as opposed to moderate, grammatical-class unspecific impairment in another—De Renzi and Di Pellegrino, 1995).

Within this general framework, our meta-analysis does find some clusters that are specific for grammatical class consistently across studies. Particularly in consideration of the fact that some of these clusters sit in areas that have gone unnoticed in previous research, it is worth taking a close look.

By means of the clustering procedure, noun-specific clusters were identified in the left angular gyrus, the left inferior parietal lobule and the right inferior frontal gyrus, pars triangularis. Given that the vast majority of the peaks in these clusters come from lexical decision, picture naming, and semantic judgment tasks, it is likely that these areas underlie lexical-semantic processing, possibly word identification and retrieval. These data further confirm the implications in the previous paragraphs, i.e., that strong embodied theories of concept representation are not supported by neurolinguistic evidence on noun and verb processing. These theories would lead one to expect visuo-motor cortices to underlie lexical and semantic processing of nouns, whereas the noun-specific clusters identified in this study are located outside those areas. Tool nouns (e.g., screwdriver, whistle) would have been a perfect test case as they are clearly related to specific motor patterns; however, activation peaks for these nouns were so rare in our data set (only 30 out of a total of 454 noun peaks) that it was not possible to apply the clustering algorithm to them alone. Nevertheless, they could well have clustered in, say, the primary motor area or posterior parietal areas had they been *consistently* located there; but in fact they did not cluster at all—only 1 of those 30 peaks is included in a noun cluster—, which indicates that they were scattered over different brain regions.

One cluster, located in the posterior part of the right middle temporal gyrus, turned out to be predominantly associated with verb processing; action-related activation in this brain region is frequently reported in the literature though its contribution is for some reason neglected. It is only in recent years that attention has been focused on the right posterior middle temporal gyrus during action processing (Kable et al., 2005; Tettamanti et al., 2005; Assmus et al., 2007; Deen and McCarthy, 2010). Assmus et al. (2007), for example, explored the neural activations associated with a familiarity judgment on pictures representing whole-body actions (e.g., dancing) vs. manipulable objects (e.g., telephone) and non-manipulable objects (e.g., motorway), observing increased bilateral activation in the middle temporal gyrus, the inferior and superior parietal cortex, and the premotor cortex. However, their study did not involve explicit linguistic processing, and so these areas might simply reflect the activation of action-related, human body representation. This interpretation is supported by the fact that the posterior part of the superior temporal gyrus is often associated with sensory-motor integration (e.g., Bangert et al., 2006) and is anatomically contiguous to the visual area MT [*x* = 45.5 (8.1); *y* = −65.9 (7.9); *z* = −0.9 (6.5); Mendola et al., 1999]. It could thus be speculated that visuo-motor processing and the sensorimotor attributes of actions may have represented the phylogenetic and ontogenetic “point of entry” for the development of a more complete action knowledge, which might have evolved gradually into a more general verb knowledge [for a similar argument on different brain areas, see Aggujaro et al. (2006) and Berlingeri et al. (2008); see also Watson and Chatterjee (2011), for a general formulation of the “point of entry” theory]. In light of this hypothesis it is intriguing that a right, and not left, hemisphere cluster in this area turned out to be associated with verbs: the two posterior middle temporal clusters identified by the algorithm in the left hemisphere contained the same quantity of noun and verb peaks. This could be explained by the fact that most studies investigated tool nouns, thus inducing activation to the left posterior temporal and inferior parietal regions, typically associated with tool use.

This hypothesis provides a certain degree of support to weak embodied theories, which simply see abstract representations as related to their visuo-motor counterpart. Verb representations would be linked to action-related, human body information, which, however, would by no means constitute the core of verb representations; these latter have their own stance *independently* of motoric information, and relate to it through the mediation of higher-level, modality independent neural systems (e.g., Hagoort, 2005; Bedny and Caramazza, 2011; van Ackeren et al., 2012).

## Conclusion

The meta-analysis described in this paper has confirmed that the neuroimaging evidence obtained so far on noun and verb processing does not indicate a great deal of grammatical class specificity in the brain, at least at the spatial resolution normally allowed by imaging experiments: most of the brain areas that have been considered as associated with noun- and verb-processing are shown to include a statistically indistinguishable quantity of noun and verb peaks, *if all the imaging studies on this issue are considered together*. These data are at odds with embodied theories of verb representation, in both the weak and strong variants, and also with the widely held account that verb processing relies on frontal areas and noun processing is based on temporal regions. Instead, these results are coherent with the idea that the neural circuits responsible for verb and noun processing are not spatially segregated in different brain areas, but are strictly interleaved with each other in a mainly left-lateralized fronto-temporo-parietal network (26 of the 32 clusters identified by the algorithm lie in that hemisphere), which, however, also includes right-hemisphere structures (Liljeström et al., 2009; Sahin et al., 2009; Crepaldi et al., 2011). In this general picture, there are indeed brain regions where noun and verb circuits cluster together so as to become spatially visible to fMRI and PET in a replicable manner, but they are limited in number and are probably located in the periphery of the functional architecture of the neural structures responsible for noun and verb processing.

### Conflict of interest statement

The authors declare that the research was conducted in the absence of any commercial or financial relationships that could be construed as a potential conflict of interest.

## Footnotes

^1^Incidentally, the role of cognitive demand, which we believe to be the second most important confounding factor in this literature, will not be investigated explicitly in this paper; this is due to the fact that this factor is virtually impossible to quantify operationally. However, because cognitive demand is highly correlated with task, we are confident that at least part of its impact is indeed taken up in this work.

^2^GingerAle analyses were run over the entire dataset of foci (i.e., noun and verb peaks were considered together) in order to create a statistical probability map comparable to the outcome of the hierarchical clustering algorithm.
